# Plant-Based Fermented Beverages: Nutritional Composition, Sensory Properties, and Health Benefits

**DOI:** 10.3390/foods13060844

**Published:** 2024-03-10

**Authors:** Belén Hidalgo-Fuentes, Edgar de Jesús-José, Anselmo de J. Cabrera-Hidalgo, Ofelia Sandoval-Castilla, Teodoro Espinosa-Solares, Ricardo. M. González-Reza, María L. Zambrano-Zaragoza, Andrea M. Liceaga, José E. Aguilar-Toalá

**Affiliations:** 1 Licenciatura en Ciencia y Tecnología de Alimentos, División de Ciencias Biológicas y de la Salud, Universidad Autónoma Metropolitana, Unidad Lerma, Av. de las Garzas 10. Col. El Panteón, Lerma de Villada 52005, Estado de Mexico, Mexico2193039504@correo.ler.uam.mx (E.d.J.-J.); 2 TecNM-Instituto Tecnológico Superior de Tlatlauquitepec (ITSTL), Carretera Federal Amozoc-Nautla km 122+600 Almoloni, Tlatlauquitepec 73907, Puebla, Mexico; anselmo.cabrera@tlatlauquitepec.tecnm.mx; 3 Departamento de Ingeniería Agroindustrial, Universidad Autónoma Chapingo, Texcoco 56230, Estado de Mexico, Mexico; 4 Agricultural Research and Extension Center, Southern University, Baton Rouge, LA 70813, USA; 5 Laboratorio de Procesos de Transformación y Tecnologías Emergentes de Alimentos-UIM, FES-Cuautitlán, Universidad Nacional Autónoma de México, Cuautitlán Izcalli 54714, Estado de Mexico, Mexicoluz.zambrano@unam.mx (M.L.Z.-Z.); 6 Protein Chemistry and Bioactive Peptides Laboratory, Department of Food Science, Purdue University, 745 Agriculture Mall, West Lafayette, IN 47907, USA; 7 Departamento de Ciencias de la Alimentación, División de Ciencias Biológicas y de la Salud, Universidad Autónoma Metropolitana, Unidad Lerma, Av. de las Garzas 10. Col. El Panteón, Lerma de Villada 52005, Estado de Mexico, Mexico

**Keywords:** dairy milk alternatives, plant-based beverages, functional foods, fermentation technology, health benefits

## Abstract

Plant-based beverages have gained consumers’ attention for being the main substitutes for dairy milk, especially for people with lactose intolerance, milk allergies, and a prevalence of hypercholesterolemia. Moreover, there is a growing demand for a more sustainable diet and plant-based lifestyle due to concerns related to animal wellbeing, environmental impacts linked to dairy production, and the rising cost of animal-derived foods. However, there are some factors that restrict plant-based beverage consumption, including their nutritional quality and poor sensory profile. In this context, fermentation processes can contribute to the improvement of their sensory properties, nutritional composition, and functional/bioactive profile. In particular, the fermentation process can enhance flavor compounds (e.g., acetoin and acetic acid) while decreasing off-flavor components (e.g., hexanal and hexanol) in the substrate. Furthermore, it enhances the digestibility and bioavailability of nutrients, leading to increased levels of vitamins (e.g., ascorbic acid and B complex), amino acids (e.g., methionine and tryptophan), and proteins, while simultaneously decreasing the presence of anti-nutritional factors (e.g., phytic acid and saponins). In contrast, plant-based fermented beverages have been demonstrated to possess diverse bioactive compounds (e.g., polyphenols and peptides) with different biological properties (e.g., antioxidant, anti-inflammatory, and antihypertensive). Therefore, this article provides an overview of plant-based fermented beverages including their production, technological aspects, and health benefits.

## 1. Introduction

Nowadays, plant-based beverages have gained interest from the scientific community, food industry, and consumers as milk alternatives. The main reasons include that they are a good alternative for people with lactose intolerance, milk allergies, and a prevalence of hypercholesterolemia [1]. In addition, a wide sector of the population is seeking a more sustainable diet, moving towards a more plant-based lifestyle (i.e., vegetarianism, veganism, and flexitarianism); also relevant are the growing ethical concerns related to animal welfare, the negative environmental impacts associated with dairy production, and the rising prices of animal-derived foods [1,2,3]. In particular, plant-based beverages can be considered to be sustainable food systems because they potentially require less resources (e.g., less water and land use) and generate a lower carbon footprint compared to dairy milk production. For example, it is estimated that the carbon footprint of milk production is around 3.2 kg of CO_2_ equivalent per liter of milk produced, whereas the carbon footprint of different plant-based beverages can range from 0.7 to 1.2 kg of CO_2_ equivalent per liter of beverage produced [4,5,6,7,8]. The considerations stated above are supported by the fact that the worldwide market of plant-based beverages is expected to grow by 15% (Compound Annual Growth Rate) annually from 2023 to 2028 [9]. In contrast, some regional markets have experienced a decline in fluid dairy milk sales. In this context, the estimated US fluid milk sales of total conventional fluid milk products had decreased 6.5% by April 2023 from a year earlier [10]. This information suggests that plant-based beverages have strong market potential with expected full market growth and a clear consumer target.

Plant-based beverages are defined as fluids that result from water extraction processes of plant material through their breakdown (size reduction) and/or homogenization [1,11,12]. Generally, the resulting particles have a size distribution ranging from 5 to 20 μm, allowing these water-soluble extracts of plant material to resemble bovine milk in appearance [1,12]. From a physicochemical perspective, plant-based beverages are colloidal suspensions or emulsions of dispersed plant material [13]. Normally, plant-based beverages are characterized as oil-in-water (O/W) emulsions, where oil is the dispersed phase and water is the aqueous/continuous phase [14,15]. This type of emulsion allows the imitation of some characteristics found in dairy milk, which is also an O/W emulsion, such as appearance, consistency, stability, mouthfeel, and taste [14,16,17]. In dairy milk, the emulsifier agents are phospholipids and milk proteins [18], while for plant-based beverages these can be biosurfactants, phospholipids, proteins, and polysaccharides that are naturally present in the plant matrix or added as additives during beverage production [15,19]. It is important to note that while these types of beverages are also referred to as “milk”, such as plant milks, vegetable milks, vegetable/plant-based milks, or non-dairy milks, this seems to not be permitted according to the legislation in many countries. For example, in the United States, the Food and Drug Administration (FDA) had defined milk as the lacteal secretion obtained from healthy cows [20]. However, in 2023, the FDA provided a new guidance for plant-based beverages to be called “milk”. According to this guidance, companies are also encouraged to voluntarily include extra nutrition labels that note when the plant-based beverages have lower levels of nutrients than dairy milk (e.g., vitamin D, calcium, and magnesium) [21]. In 2020, European Union regulations stated that the term “milk” could only be used to market and advertise products derived from animal milk, thus banning dairy-related terms for plant-based alternatives. Similarly, in Mexico, the Mexican Official Standard (NOM-155-SCFI-2012 [22]) mentions that milk is derived exclusively from the mammary secretion obtained from cows. Thus, in Mexico, plant-based beverages cannot be called milks and are referred to as “beverages” or “drinks”.

**Figure 1:**
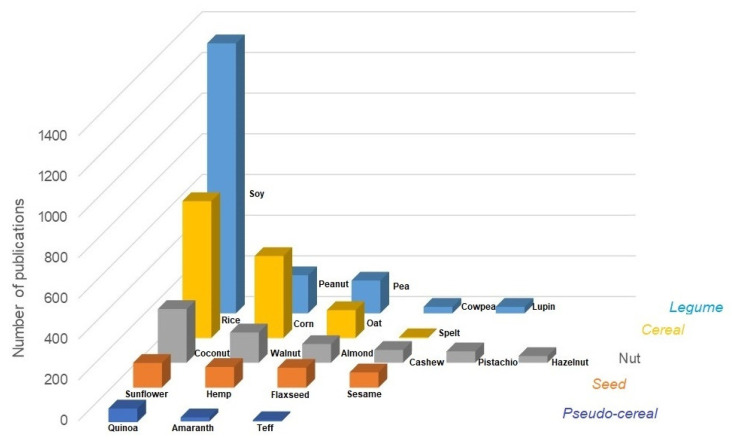
Comparative chart of recent publications related to plant-based beverages. The search was conducted on the Web of Science Core Collection database (Clarivate analytics, USA) on December 2023. Original scientific studies dating from January 2018 to November 2023 were included. The main search terms used were the “name” of the plant-based beverage AND “milk” (e.g., soy milk OR soymilk) OR “-based beverage” OR “extract”, consulting scientific studies published in English.

The growing interest in plant-based beverages is highlighted in Figure 1, which shows the literature accumulating ca. 4100 publications, available from 2018 to 2023, related to plant-based beverages from any of the five categories: legume, nut, cereal, pseudo-cereal, and seeds. Of these publications, a total of 247 are directly related to fermented beverages. Soy-, rice-, corn-, coconut-, and peanut-based beverages have the highest number of publications (>190), with soy-based beverages being noticeably the most studied (ca. 1300 publications). These beverages have been mainly evaluated for their nutritional characterization, off-odor compounds, and antinutrient profile, as well as for starch, lipid, and protein extraction [23,24,25], and little attention has been paid to their fermentation [26,27,28]. Nut- and cereal-based beverages have also gained popularity in the last 5 years as they are considered rich in nutrients and bioactive compounds [29,30].

Initial research on plant-based beverages focused attention towards soy-based beverages as the main alternative to dairy milk, due to their nutritive value, as well as higher protein content and lower price [1,31]. Nevertheless, in recent years, the use of other plant materials such as legumes, nuts, seeds, cereals, and pseudo-cereals have been explored in the formulation of plant-based beverages [32]. In addition, mixing two or more plant materials to produce plant-based beverages could add or complement the nutritional value and at the same time produce a novel product [12]. One way to innovate and meet the needs of consumers is the elaboration of plant-based fermented beverages [33], which not only improves their sensory properties and nutritional composition but also offers the opportunity to develop functional foods with health benefits. This gives a whole new set of possibilities for novel beverages with different tastes, aromas, flavors, textures, mouthfeels, and nutritive characteristics. Therefore, this review aims to provide an in-depth exploration of the main types and characteristics of plant-based beverages, while emphasizing the significant influence of the fermentation process on their sensory properties, nutritional compositions, and the biological activity associated with their health benefits.

## 2. Plant-Based Beverages: Types and Characteristics

Plant-based beverages are growing in interest and acceptance from consumers, while also being visualized as promising and viable alternatives to dairy-based products [34]. Although the soy-based beverages were the first produced, at the present time, there are a wide variety of these beverages from various plant sources [1,31]. In accordance with the above, plant-based beverages can be classified into five main categories based on their source [1,32]:

1. Legume beverages: soy, peanut, pea, lupin, and cowpea;
2. Nut beverages: almond, coconut, hazelnut, pistachio, walnut, and cashew;
3. Cereal beverages: oat, rice, corn, and spelt;
4. Pseudo-cereal beverages: quinoa, teff, and amaranth;
5. Seed beverages: sesame, flaxseed, hemp, sunflower.

In search of dairy alternatives, both the scientific community as well as the food and pharmaceutical industries have investigated various plants matrices such as legumes, cereals, nuts, pseudo-cereals, and seeds for their functional properties because of their content of nutritional and health compounds [16]. In this context, legume-based beverages are characterized as having a balanced composition, since these beverages have a protein content (ca. 3–4%) similar to cow´s milk (ca. 3.5%) [35]. In this group of beverages, soy is the most representative and most consumed legume-based beverage. Around 2000 years ago, this plant was the initial source utilized in China to produce a substitute for milk [16]. Among these legume-based beverages, the peanut-based beverage introduces a roasted and nutty taste, while the pea-based beverage offers a slightly sweet flavor [36]. In contrast, nut-based beverages encompass a variety of plant-based drinks derived from different dry fruits composed of a hard inedible husk and a seed, which are characterized by their high lipid (ca. 3–5%) and protein (ca. 0.8–1.3%) content [30,37,38]. Almond milk, one of the most widely consumed nut-based beverages, has a mild taste and versatility of use. Coconut milk gives off a characteristic tropical flavor and creamy texture, making it a favorite in various culinary applications [39]; hazelnut milk introduces a rich and slightly sweet profile; and pistachio milk offers a unique and nutty taste [40].

On the other hand, cereal-based beverages are characterized as having a protein and lipid content ranging from 0.3 to 3% and 2.3 to 7%, respectively, in addition to their naturally subtle sweetness [29,37]. In particular, in this group, oat- and rice-based beverages are praised for their versatility in coffee, cooking, and baking applications [41]. For example, oat-based beverages are characterized by their noticeable dietary fiber (3–4%) content [1,38], while rice-based beverages are rich in carbohydrates (ca. 9%) [42]. In contrast, beverages made from corn and spelt are valued for their sweet–sour taste and thin yet gritty–smooth consistency [43]. Similarly, pseudo-cereal beverages include quinoa, teff, and amaranth, which can have a variety of flavors from nutty and earthy to slightly sweet or bitter [44,45,46]. Principally, these beverages possess a range of protein and lipid contents between 0.5 and 3.5% and 0.6 and 3%, respectively. Meanwhile, seed-based beverages including sesame, flaxseed, hemp, and sunflower offer delicate nutty and earthy undertones [45,46]. In particular, seed-based beverages are characterized for their variable lipid (ca. 1–7%) and protein (ca. 1–5%) content [37,38].

In general, plant-based beverages have several advantages and disadvantages compared with dairy milk. For example, these beverages do not contain lactose, milk protein, or cholesterol, which is beneficial for people with lactose intolerance, milk allergies, and a prevalence of hypercholesterolemia, respectively [47,48]. Regarding this last matter, plant-based beverages are low in saturated fat, while containing more polyunsaturated fatty acids, which are associated with a lower incidence of chronic diseases [49]. Additionally, in contrast to dairy milk, plant-based beverages contain fiber [33,49].

Some disadvantages of plant-based beverages are associated mainly with their low micronutrient content as well as lower protein quality [38,50]. In this context, most plant-based beverages tend to have lowers levels of essential micronutrients such as vitamins (i.e., B complex, B_6_, K, E, and folate), magnesium, potassium, and zinc. In addition, these beverages are characterized by their low protein content, except for specific cases such as soy protein, as well as a lack of all the essential amino acids in the optimal ratios required by the human body in comparison with dairy milk [37,38,50]. For example, the protein quality reported as the Digestible Indispensable Amino Acid Score (DIAAS) is low for rice protein isolate (ca. 37%) and relatively high for soy protein (ca. 90%), while dairy milk possess a DIAAS of 100% [50]. In addition, they can contain antinutrient factors such as inositol phosphates, phytic acid, and trypsin inhibitors, as well as protein allergens [13,50]. Another concern with plant-based beverages is that they can have a negative impact on the sensory characteristics of these beverages, including unpleasant aromas and flavors and poor texture [51]. For example, soy beverages tend to have a beany flavor and seed-based beverages can present bitterness [1,26]. In particular, in legume-based beverages, the compounds hexanal and hexanol have been identified as responsible for the beany flavors [23,26]. Similarly, Pérez-González et al. (2015) [52] identified several aldehydes (e.g., pentanal and hexanal) and alcohols (e.g., heptanol) in an almond beverage. These compounds are formed via lipid oxidation processes and are responsible for the off-flavor components in plant-based beverages. Some compounds such as alkaloids, phenols, saponins, cyanogenic glycoside, flavonoids, terpenes, and glucosinolates are known to impart bitterness and astringency to plant-based beverages [26,53]. The content and diversity of these compounds depended on the plant source, which results in a difference in acceptability for these beverages. In this context, Jeske et al. [54] conducted a sensory acceptance test of commercial plant-based beverages (i.e., oat, rice, hemp, almond, soy, and lentil) and found that the most accepted beverages were oat and rice, while the least accepted was hemp.

In this sense, for the case of plant-based beverages, the fermentation processes could be a useful technology that contributes to the improvement of sensory properties and nutritional composition, similar to what occurs with other fermented foods such as cheese, yogurt, and bread [26,55].

## 3. Impact of Fermentation on Nutritional Composition, Sensory Properties, and Bioactive Profile of Plant-Based Beverages

Fermentation has been used since ancient times for preserving or preparing new foods and beverages [56] and improving their flavor and texture [57]. It is an enzyme-driven process facilitated by microorganisms, breaking complex macromolecules into simpler ones [28,55,56]. This process can produce various compounds such as proteins, amino acids, fatty acids, vitamins, acetic acid, and volatile compounds, imparting different flavors, aromas, and textures to foods such as cheese, yogurt, kefir, and bread [55,57,58]. Moreover, it is cost-effective and operationally easy, making it a feasible option for food processing [59]. In the case of plant-based beverages, the fermentation process can vary depending on the composition of the specific plant matrix being fermented and the microorganisms involved, for example, lactic acid and alcoholic fermentations, or in some cases, a combination of both. Other types involve, to a lesser extent, acetic acid fermentation [2,60]. Typically, lactic acid bacteria such as *Lactobacillus* and *Streptococcus* participate in lactic acid fermentation, where carbohydrates are converted into lactic acid that imparts sensory properties and also helps preserve the fermented product. In contrast, during alcoholic fermentation, yeast such as *Saccharomyces cerevisiae* participates in the conversion of carbohydrates into alcohol (e.g., ethanol) and carbon dioxide [17,32,57,60].

**Figure 2:**
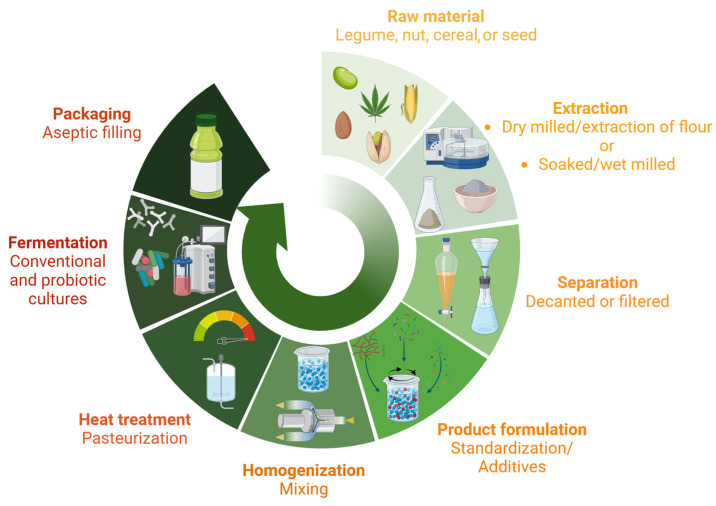
General diagram of the production process for plant-based fermented beverages. Common procedures were adapted from Alexandre et al. [62] and Gil-Serna et al. [63]. Created using Biorender.com (accessed on 11 December 2023).

The typical production process for plant-based beverages starts with water extraction of the raw material either via soaked/wet-milling or dry-milling and extraction of powder/flour. This is followed by the separation of solids via filtration, product formulation using different additives and ingredients, homogenization, heat treatment to inactivate the endogenous enzymes and microbial load followed by the fermentation process, and finally product packaging (Figure 2). In the case of plant-based beverages, it has been reported that fermentation contributes to the improvement of their sensory properties, safety quality, nutritional composition, and bioactive profile [26,27,28]. The above is attributed mainly to microorganisms that utilize nutrients during their growth in the matrix of the plant-based beverages, which lead to changes in the components and their concentrations [61].

### 3.1. Impact on Nutritional Composition

The nutritional composition of plant-based beverages is improved by the fermentation processes by enhancing the digestibility and availability of nutrients, as well as producing nutritional factors such as vitamins. In addition, this biochemical process can help in the elimination of anti-nutritional factors, which is beneficial for enhanced nutrient bioavailability. During the fermentation process, enzymes and microorganisms break down complex macromolecules (i.e., proteins and carbohydrates) present in the plant matrix into simpler forms. In the case of proteins, this process can lead to improved protein digestibility as proteins are partially hydrolyzed into polypeptides, oligopeptides, and free amino acids, making them more accessible and easier for the body to absorb [64]. In addition, the amino acid composition of the plant-based beverages is also modified during fermentation because some amino acids are synthesized or released from peptides by microorganisms, leading to changes in the overall amino acid profile. The resulting amino acid profile can vary depending on the initial protein composition of the specific plant matrix being fermented and the microbial enzymes (e.g., cell-envelope proteinases and intracellular peptidases) involved [65]. For example, lactic acid bacteria contain cell-envelope proteinases that start the proteolysis of plant proteins, breaking them down into oligopeptides that are then used by cells through specific peptide-transport systems or degraded into small peptides and free amino acids via the collective action of various intracellular peptidases [66]. Fermentation can also enhance mineral bioavailability by decreasing the presence of anti-nutritional factors such as phytic acid, saponins, condensed tannins, and trypsin inhibitors, among others, which can bind to minerals and hinder their absorption [67,68]. During fermentation, some microorganisms can produce enzymes that degrade anti-nutritional factors or they can modify them as part of their microbial metabolism. Some changes in pH can also affect the stability of these factors [67,69]. For example, microbial phytases and tannases, derived from lactic acid bacteria or yeast, can degrade phytates and tannins, respectively [69,70,71]. Similarly, it has been reported that some lactic acid bacteria can metabolize oxalates, reducing their concentration in the final fermented product [72].

In this sense, Pontonio et al. [73] reported that a fermented beverage prepared using a mix of rice, lentil, and chickpea flours showed a higher biological value, protein efficiency ratio, essential amino acid index, and total of free amino acids compared to the unfermented beverage. In particular, the authors found that the in vitro protein digestibility increased from 67 to 79% after the fermentation process. In addition, lower contents of the antinutritive factors (i.e., phytic acid, condensed tannins, saponins, and raffinose) were found in the fermented beverage. Likewise, Vila-Real et al. [74] reported that a fermented millet-based, yoghurt-like beverage showed a higher content of threonine, arginine, glutamine, and gamma-aminobutyric acid (GABA) compared to the unfermented beverage. The authors reported that protein digestibility increased from 25 to 64% after fermentation. In another study, Sanni et al. [75] reported that pea-based fermented beverages showed higher protein, total ash, calcium, potassium, phosphorus, lysine, methionine, tryptophan, riboflavin, niacin, and thiamine than their unfermented counterparts. The antinutritive factors (i.e., phytic acid and trypsin inhibitor) in this fermented beverage decreased after the fermentation process. Likewise, Rekha and Vijayalakshmi [76] reported that the mineral bioavailability (i.e., calcium, magnesium, and zinc) and vitamin B complex (i.e., thiamin, riboflavin, and niacin) increased in soy-based fermented beverages compared with the unfermented versions. In addition, the authors observed a decrease in phytic acid in the final fermented beverage. Other studies [77] found that a soy-based beverage fermented by *Streptococcus thermophilus* 14085 and *Bifidobacterium infantis* 14603 showed a lower content of saponins and phytates. Hou et al. [78] reported that soy-based beverages fermented by *Bifidobacterium longum* B6 and *B. infantis* CCRC 14633 showed a significant increase in protein, thiamin, and riboflavin after 48 h of fermentation. On the other hand, using a Caco-2 cell model, Bernat et al. [79] described that the fermentation of an almond-based beverage using different potentially probiotic bacteria increased the bioavailability of dietary iron. Santos et al. [80] reported that a peanut–soy beverage fermented using six different lactic acid bacteria (LAB) strains, including probiotic strains, and yeasts, increased the amount of total amino acids compared with the unfermented beverage. The authors also reported that, when co-cultured, the LAB strains were more efficient at utilizing carbohydrates and releasing metabolites compared to the single culture fermentations. In a related study, using a liquid formulation of the serine–endoprotease subtilisin (derived from *Bacillus subtilis*) on soy pulp (okara) water extract (a soy by-product beverage) resulted in higher extracted proteins [81]. Likewise, Karovičová et al. [82] reported an increase in protein content by ca. 40% after fermentation of a quinoa-based beverage. Similarly, Jeske et al. [83] also reported that a quinoa-based beverage treated using different enzymes increased the protein content by 1.5-fold compared to the control (no enzyme added) beverage.

### 3.2. Impact on Sensory Properties

With regard to the sensory characteristics of plant-based beverages, the fermentation processes seem to increase the production of flavor compounds such as organic acids and volatile metabolites. For instance, Zheng et al. [84] found that the sensory properties of a soy-based beverage fermented by *Lactobacillus harbinensis* M1 were improved by the production of 2,3-butanedione and acetoin, which impart a buttery aroma. The authors reported that, after fermentation, the hexanal content decreased to undetectable levels. Similarly, soy-based beverages supplemented with okara flour and fermented by *Lactobacillus acidophilus* LA3 increased significantly (*p* < 0.05) in their content of organic acids (e.g., lactic and acetic acids) and also showed enhanced sensory acceptability [85]. Likewise, Nissen et al. [86] reported a shift in the aromatic profile of hemp-based beverages fermented by probiotic bacteria (*Lactobacillus fermentum*, *Lb. plantarum*, and *Bifidobacterium bifidum*), generating 2,3-butanedione and acetoin, which conferred a pleasant buttery taste to the drinks and improved the overall mouthfeel. In contrast, Menezes et al. [87] reported that maize-based beverages fermented by probiotic yeast and lactic acid bacteria (e.g., *Lactobacillus paracasei* LBC-81, *Saccharomyces cerevisiae* CCMA 0731, *S. cerevisiae* CCMA 0732, and *Pichia kluyveri* CCMA 0615) produced 70 volatile compounds, including acids, alcohols, aldehydes, esters, and ketones, with lactic and acetic acids being the main metabolites detected. In the same way, Horáčková et al. [88] reported that a soy-based beverage fermented by a yoghurt culture (YC-381) in combination with two probiotic bacteria (*Bifidobacterium animalis* subsp. *lactis* BB 12 and *Bifidobacterium bifidum* CCDM 94) produced considerable amounts of lactic acid, acetic acid, and acetaldehyde, which provide the typical aroma and flavor of yoghurt. Similarly, Demarinis et al. [89] determined the sensory characteristics of various plant-based beverages (i.e., lupin and pea) fermented by specific lactic acid bacteria strains. The lupin- and pea-based beverages underwent an in-depth sensory evaluation using a simplified check-all-that-apply (CATA) method, displaying sensory notes such as cooked ham, almonds, and sandalwood. Also, diverse cereal-based beverages fermented using different lactobacilli strains exhibited different sensory characteristics, with fermented beverages with a higher acetaldehyde content having better sensory acceptability [90].

On the other hand, other strategies to improve the sensory properties of this type of plant-based fermented beverage include the blending of different plant sources or the addition of fruit pulps. For instance, studies show that mixing two plant-based beverages (i.e., soy and almond, 50:50% *v*/*v*) significantly improved (*p* < 0.05) the sensory properties (e.g., color, flavor, mouthfeel, and overall acceptability) of the final beverage compared with the 100% fermented soy-based beverage (nine-point hedonic scale, *n* = 10) [91]. Similar results were obtained by Antoine et al. [92] by mixing 20% *v*/*v* of cashew nut (*Anacardium occidental*) and 80% *v*/*v* of soy (*Glycine max* L.) beverages, which increased their sensory properties (e.g., sweetness and aroma) according to the descriptive analysis (N = 10) of those beverages fermented using probiotic potential strains compared with those fermented using conventional yoghurt strains. The addition of fruit pulps or flavoring agents can help improve the acceptability of the fermented beverages. Karovičová, Kohajdová, Minarovičová, Lauková, Greifová, Greif, and Greif [82] reported a significant (*p* < 0.05) improvement in the acceptability of a quinoa-based fermented beverage when a raspberry syrup (10% *v*/*v*) was added (ca. 91% acceptability compared to the non-supplemented beverage, N = 11). Similarly, a rice-based fermented beverage enriched with 10% *v*/*v* of cactus pear (*Opuntia* spp.) and 10% *v*/*v* of physalis (*Physalis peruviana*) fruit pulps significantly enhanced (*p* < 0.05) the overall acceptability (on a seven-point hedonic scale, N = 10) compared with the non-enriched rice-based fermented beverage [93]. Likewise, Sanni, Onilude, and Adeleke [75] reported significantly (*p* < 0.05) more overall acceptability (on a nine-point hedonic scale, N = 9) of pea-based fermented beverages with added banana and strawberry flavors than the control (unflavored) fermented beverage. The addition of fruit pulps can enhance the sensory acceptability of the final fermented product because they provide natural sweetness. In addition, the fermentation of the simple sugars present in these fruit pulps can generate new sweet taste profiles [94,95]. Fruits pulps also contain aromatic compounds (e.g., aldehydes), and their consistency can improve the mouthfeel of the plant-based fermented beverages [95,96].

### 3.3. Impact on Bioactive Profile

During the fermentation of plant-based beverages, a diverse group of bioactive compounds and metabolites are generated. In particular, microorganisms used for the fermentation process hydrolyze oligosaccharides and proteins present in plants that are not commonly digested in the human gut [28,97]. According to Table 1, the main bioactive compounds and metabolites generated and/or liberated from the plant matrix of the beverages include polyphenols (e.g., flavonoids), vitamins (e.g., ascorbic acid and tocopherol), isoflavone aglycones (e.g., genistein, daidzein, and glycitein), bioactive peptides, amino acids (e.g., gamma-aminobutyric acid, alanine, and arginine), and organic acids (e.g., lactic acid). In addition, in the majority of cases, plant-based fermented beverages are produced from soy and, to a lesser extent, from almond, oat, flaxseed, rice, cashew, coconut, and hemp, among others (Table 1). This may be due to the fact that, among the above-mentioned plant-based beverages, soy has a higher and more balanced nutrient content comparable to that of cow´s milk (e.g., protein and fat) [98] and is one of the most accepted plant-based beverages [54]. Various other sources have also been used to produce these fermented beverages, but to a relatively minor degree, such as lupin, lentil, chickpea, camelia, and apricot, to name a few.

Research has determined the presence and/or content of peptides, total polyphenols, flavonoids, and tocopherol in soy-based fermented beverages, which increase during the fermentation time [82,99,100,101], and their production/profile depends on the metabolic activity of microorganisms involved [61]. Some of these studies reported a relationship between the increase in specific bioactive compounds and the bioactivity tested. For example, Tonolo, Moretto, Folda, Scalcon, Bindoli, Bellamio, Feller, and Rigobello [99] reported that, after fermentation, the content of peptides and polyphenols as well as the antioxidant activity of a soy-based fermented beverage increased during storage time (weeks). Similarly, Undhad Trupti, Das, Solanki, Kinariwala, and Hati [100] reported that during the fermentation of a soy-based beverage, bioactive peptides were released with antihypertensive activity towards the inhibition of angiotensin-converting enzyme (ACE). Likewise, de Queirós, de Ávila, Botaro, Chirotto, Macedo, and Macedo [101] and Karovičová, Kohajdová, Minarovičová, Lauková, Greifová, Greif, and Greif [82] found that soy-based and quinoa-based beverages, respectively, increased in their total polyphenolic content and antioxidant activity. In particular, in soy-based fermented beverages, isoflavone aglycones (daidzein, genistein, and glycitein) have been identified. To obtain these aglycone forms, soy isoflavones should be converted from their glycosylated (e.g., daidzin, genistin, and glycitin) to aglycones forms through the action of β-glycosidase from microorganisms used to fermented the soy-based beverages [102,103]. There are some reasons for the interest in transforming glycosylated isoflavones into their aglycone forms because this increases their bioavailability, their bioactivity/health benefits, and facilitates their transformation into other bioactive secondary metabolites (e.g., equol) [102]. The above-mentioned isoflavones are known to exhibit antioxidant, chemoprotective, osteogenetic, and anti-inflammatory properties [61,103].

In other studies, the presence of polyphenols, flavonoids, and ascorbic acids in flaxseed- [104] and lupin-based [105] fermented beverages showed antioxidant properties. Similarly, fermented beverages derived from an almond–soy blend [106] and hemp [107] demonstrated antioxidant activity as well as a noticeable content of total polyphenols and flavonoids. In addition, other bioactive compounds such as GABA were found in fermented beverages derived from a rice–chickpea blend [73], rice [108], apricot seed [109], and coconut [110]. These fermented beverages showed antioxidant and antimicrobial activities, as well as angiotensin-converting enzyme (ACE), lipase, α-amylase, and α-glucosidase inhibition properties. These properties were attributed to the presence of GABA and other compounds such as polyphenols and lactic acid [73,108,109,110]. In contrast, other studies have demonstrated the release of bioactive peptides after fermentation from proteins in soy [100,111], flaxseed [112], coconut [113], and oat [114] beverages, which presented antioxidant and antimicrobial properties as well as ACE inhibition [91,100,113,114].

**Table 1:** Bioactive compounds and their bioactivities present in fermented plant-based beverages.

Fermented Plant-Based Beverage	Microorganisms	Bioactive Compounds	Bioactivity	References
Soy (*Glycine max*)	*Lactobacillus delbrueckii* subs. *Bulgaricus* and *Streptococcus thermophilus*	Polyphenols, tocopherol, and peptides	Antioxidant activity	[99]
Soy (*Glycine max*)	*Lactobacillus rhamnosus* CRL981	Isoflavone aglycon	Antioxidant activity	[115]
Soy (*Glycine max*)	*Bacillus subtilis* MTCC5480 and *Bacillus subtilis* MTCC1747	Peptides and polyphenols	Antioxidant activity	[111]
Soy (*Glycine max*)	*Bacillus subtilis* 10160	Polyphenols and flavonoids	Antioxidant activity and ACE inhibition	[116]
Soy (*Glycine max*)	*Lactobacillus* sp. FTDC 2113, *Lactobacillus acidophilus* FTDC 8033, *Lactobacillus acidophilus* ATCC 4356, *Lactobacillus casei* ATCC 393, *Bifidobacterium* FTDC 8943, and *Bifidobacterium longum* FTDC 8643	Isoflavone aglycones (genistein)	ACE inhibition	[117]
Soy (*Glycine max*)	*Lactobacillus fermentum* M2 and *Lactobacillus casei* NK9	Peptides	ACE inhibition	[100]
Soy (*Glycine max*)	*Bifidobacterium animalis* ssp. *lactis* BLC 1, *Lactobacillus acidophilus* LA 3, *Streptococcus thermophilus* ST 066, *Lactobacillus casei* MB151 (ATCC 334), *Lactobacillus delbrueckii* ssp. *bulgaricus* MB153 (ATCC 9649), *Lactobacillus rhamnosus* MB154 (ATCC 7469), and *Lactobacillus kefiri* CBMAI21	Total polyphenols as well as isoflavone aglycones (daidzein, genistein, and glycitein)	Antioxidant activity	[101]
Soy (*Glycine max*)	*Lactobacillus curieae* CCTCC M2011381	Flavonoids	Antioxidant activity, ACE inhibition, and HMGR inhibition	[118]
Oat (*Avena sativa*)	*Lactobacillus plantarum* 22158, *Lactobacillus acidophilus* 6089, *Lactobacillus casei* 6117, *Lactobacillus delbrueckii* subsp. *bulgaricus* 57004, and *Streptococcus thermophilus* 58013	Polyphenols and flavonoids	N.D.	[119]
Flaxseed (*Linum usitatissimum*)	Commercial Kefir grains (Yoghurt-Tek^®^, Lactoferm Kefir Series, Kefir-31) and Biochem S.R.L. (Rome, Italy)	Polyphenols, flavonoids, and ascorbic acid	Antioxidant activity	[104]
Flaxseed (*Linum usitatissimum*)	*Lactobacillus plantarum* (NCDC374)	Peptides	Antioxidant activity, ACE inhibition	[112]
Sweet blue lupin (*Lupinus angustifolius* L. cv. “Boregine”)	Commercial Kefir grains (Yoghurt-Tek^®^, Lactoferm Kefir Series, Kefir-31) and Biochem S.R.L. (Rome, Italy)	Polyphenols, flavonoids, and ascorbic acid	Antioxidant activity	[105]
Rice (*Oryza sativa*)–Lentil (Lens culinaris)–Chickpea (*Cicer arietinum* L.)	*Lactobacillus plantarum* DSM33326, *Lactobacillus brevis* DSM33325, and *Lactobacillus rhamnosus* SP1	GABA	Antioxidant activity	[73]
Camelina (*Camelina sativa* L.) seed	Commercial yogurt starter culture YO 122	Polyphenols	Antioxidant activity	[120]
Baru (*Dipteryx alata* Vog.) almond	Commercial yogurt starter culture YF-L811, Christian Hansen^®^ Probiotic culture (*Lactobacillus casei* 01, Christian Hansen^®^	Polyphenols	Antioxidant activity, α-amylase inhibition, and α-glucosidase inhibition	[121]
Almond (*Prunus dulcis*)	Kefir grains	N.D.	Antimicrobial activity	[122]
Soy (*Glycine max*)–Almond (*Prunus dulcis*)	*Bifidobacterium longum* DSM 20219 and *Bifidobacterium animalis* subsp. *lactis* DSM 10140	Polyphenols and flavonoids	Antioxidant activity	[106]
Cashew nut (*Anacardium occidentale*)–Soy (*Glycine max* L.)	*Weissella paramesenteroides* TC6 and *Enterococcus faecalis* A4	N.D.	Antioxidant and anti-inflammatory activities	[92]
Coconut (*Cocos nucifera* L.)	Kefir grains	Peptides	Antimicrobial and antioxidant activities	[113]
Coconut (*Cocos nucifera* L.)	*Lactiplantibacillus plantarum* ngue16	GABA, lactic acid, alanine, and arginine	Antimicrobial and antioxidant activities	[110]
Hemp (*Cannabis sativa*)	Commercial Kefir grains (Yoghurt-Tek^®^, Lactoferm Kefir Series, Kefir-31) and Biochem S.R.L. (Rome, Italy)Commercial yogurt starter culture YO 122	Polyphenols and flavonoids	Antioxidant activity	[107]
Hemp (*Cannabis sativa*)	*Bifidobacterium longum* B 379M	Flavonoids and curcumin (added)	Antioxidant activity	[123]
Apricot seed (*Prunus armeniaca*)	Commercial Kefir culture (KF2 100 MU), Maysa Company (Istanbul, Turkey)	GABA	Antioxidant activity and ACE inhibition	[109]
Cashew (*Anacardium occidentale*)	*Lactobacillus acidophilus* TISTR 1338, *Lactobacillus casei* TISTR 390, and *Lactobacillus plantarum* TISTR 543	Polyphenols and ascorbic acid	Antioxidant activity	[124]
Oat (*Avena sativa*)	Commercial yogurt starter culture (CSL, Italy)Probiotic culture (*Lactobacillus casei* 01, Christian Hansen^®^	Peptides	ACE inhibition	[114]
Brown rice (*Oryza sativa*)	*Lactobacillus pentosus* 9D3	Polyphenols and GABA	Antioxidant activity, lipase inhibition, α-amylase inhibition, and α-glucosidase inhibition	[108]

ACE: Angiotensin I-converting enzyme; HMGR: 3-hydroxy-3-methylglutaryl-coenzyme A reductase (its inhibition suggested a cholesterol-lowering effects); GABA: gamma-aminobutyric acid; N.D.: Not determined or not reported.

**Figure 3:**
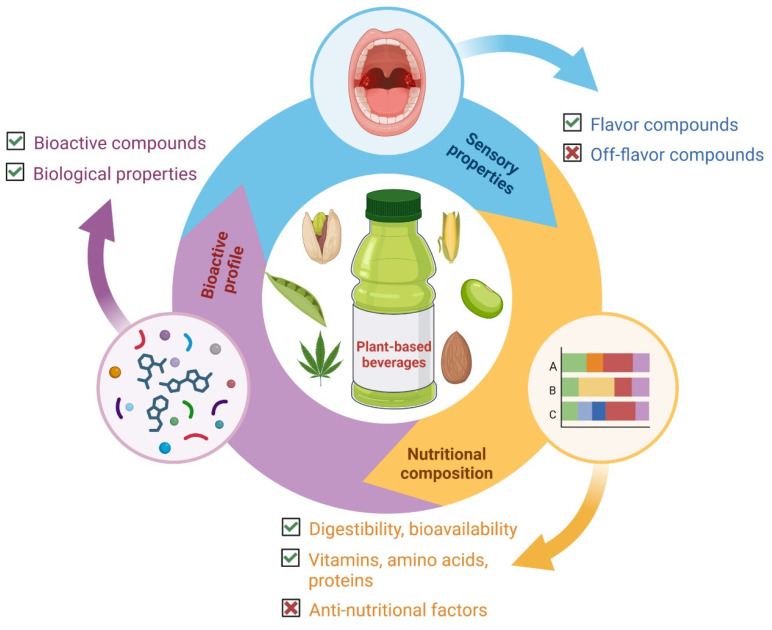
General impact of the fermentation processes on main plant-based beverage aspects. ☑ indicates increased; ⊠ indicates decreased. Created using Biorender.com (accessed on 11 December 2023).

As previously discussed, fermentation processes can improve the overall nutritional value, sensory characteristics and acceptability, and bioactive properties of plant-based beverages via the modification of their components and their bioavailability (Figure 3). In most cases, an increase in different nutritional components (e.g., proteins, amino acids, and vitamins) as well as an improvement in the digestibility of proteins and minerals bioavailability was observed. Additionally, a general decrease in anti-nutritional factors (e.g., phytic acid, condensed tannins, and saponins), which contribute to enhanced nutrient bioavailability, as found. Both observations are related to the improvement of the nutritional value of plant-based beverages as a result of the fermentation process. Similarly, the sensory properties of fermented plant-based beverages are further favored by the generation of flavor compounds during fermentation, which improve the sensory acceptability of these beverages. Likewise, the impact of fermentation on the bioactive profile is reflected because fermentation generates a diverse group of bioactive compounds/metabolites with unique biological properties that are beneficial for the health of consumers; this can contribute to the growing preference of consumers for these fermented beverages. Some mechanisms of modification of the nutritional, sensory, and bioactive profiles that occur during the fermentation process of plant-based beverages include chemical modifications (e.g., decarboxylation and hydrolysis) or the biosynthesis/release of novel compounds/metabolites during the fermentation process due to the use of nutrients as substrates and the metabolic activity of microorganisms leading to changes in the level of some constituents.

## 4. Health Benefits of Plant-Based Fermented Beverages

As mentioned in the previous section, fermented plant-based beverages contain a variety of bioactive compounds, primarily phytochemicals, that have demonstrated diverse biological properties. These properties are associated with different health benefits when these fermented products are consumed. The health effects of plant-based fermented beverages have been tested using in vivo (animal) models and clinical (human) trials, which are summarized in Table 2.

For example, Chen, Wu, Yang, Xu, and Meng [125] reported that the administration (10 mL per day for 6 weeks) of a soy-based beverage fermented by *Lactococcus acidophilus* decreased total cholesterol, triglycerides, and low-density lipoprotein cholesterol levels in the serum and liver of animals in a hyperlipidemic rat model. The authors reported an improvement in the antioxidant activities of superoxide dismutase and glutathione peroxidase, as well as increased total antioxidant capacity in blood serum. Similarly, Miraghajani, Zaghian, Mirlohi, Feizi, and Ghiasvand [126] found that the administration (200 mL per day for 8 weeks) of a probiotic-fermented soy beverage improved some oxidative stress factors, including antioxidant enzymes such as glutathione peroxidase, and glutathione reductase. Another study reported the potential antidiabetic and anti-obesity properties of a soy-based beverage (10 mL per day for 90 days) fermented using kefir grains using an animal model where rats were fed a hypercaloric high-fat, high-fructose diet. This fermented beverage was able to inhibit α-amylase and lipase enzymes in the pancreas and small intestine, while decreasing total cholesterol, LDL-cholesterol, and glucose levels in blood serum (Tiss, Souiy, Abdeljelil, Njima, Achour, and Hamden) [127].

Hu, Chen, Qian, Ye, Long, Park, and Zhao [128] reported the in vivo antioxidant and anti-aging effects of a soy-based fermented beverage (0.2 mL per day for 8 weeks) using a murine model with aging induced by D-galactose. Their results showed an increase in glutathione and total antioxidant capacity; additionally, the enzyme activities of superoxide dismutase, glutathione peroxidase, and catalase were increased in the serum, brain, and liver of animals administered the fermented beverage. In a similar study, Deeseenthum, Luang-In, John, Chottanom, and Chunchom [131] reported that rice-based fermented (150 mg dissolved in PBS per day for 10 days) beverages exhibited a in vivo antioxidant effect on a rat colitis model through the decrease in lipid peroxidation and increase in superoxide dismutase in serum. On the other hand, Aparicio-García, Martínez-Villaluenga, Frias, Crespo Perez, Fernández, Alba, Rodríguez, and Peñas [129] reported an antihyperlipidemic effect on celiac patients via the administration (200 mL per day for 6 months) of an oat-based fermented beverage. The authors observed decreased total cholesterol and triglycerides levels in the serum of treated patients. Similarly, Algonaiman, Alharbi, and Barakat [130], using a murine model, reported that, compared to the control group, oat-based fermented beverage extracts (7 mL per day for 6 weeks) were significantly (*p* < 0.05) more effective at improving serum lipid profiles in the animals. Moreover, the administration of the fermented beverage efficiently increased the amount of antioxidant enzymes and decreased lipid peroxidation levels. In another study, Wang, Wang, Han, Liang, Zhang, Bai, Yue, and Gao [134] found that the administration of fermented apple juice (10 mL per day for 4 weeks) to type 2 diabetic mice decreased fasting blood glucose and insulin levels and regulated blood lipid metabolism.

Finally, the administration of a sweet cucumber-based fermented beverage had a noticeable antihypertensive effect when using a spontaneously hypertensive rat model [133].

In summary, most of the plant-based fermented beverage studies reported in the literature were performed using in vitro assays, perhaps because in vitro studies are a more economical way to determine preliminary characterization and biological properties compared with in vivo studies. However, there is a lack of sufficient in vivo animal and/or clinical trials to determine the effectiveness of these fermented beverages with different potential beneficial effects towards humans.

## 5. Conclusions and Future Directions

Plant-based beverages have drawn attention because they are a good alternative for people with lactose intolerance, milk allergies, and a prevalence of hypercholesterolemia. Also, consumers’ demand for a more sustainable, plant-based diet has led to their rise in popularity. The fermentation processes, using different microorganisms, can improve their nutritional composition and overall sensory characteristics. However, while these beverages have been proposed as alternatives to dairy milk, they lack equal nutritional value. Although certain plant-based beverages, like those derived from soy, offer comparable nutrients to dairy milk, other strategies such as blending different plant sources, mixing with dairy milk, or supplementing with additional ingredients (e.g., fruits) or additives are recommended to enhance their overall nutritional value. In addition, the fermentation process can decrease the content of antinutritive factors, thereby preserving their nutritional quality. Research into fermented plant-based beverages faces significant challenges related to improving processing techniques, including exploring emerging non-thermal processing technologies that can ensure the safety of the product while maintaining nutritional and sensory qualities. In addition, plant-based fermented beverages have been demonstrated to have diverse compounds with different biological properties toward human health. Further characterization of these bioactive compounds, including metabolomics and proteomics, as well as animal studies and/or human clinical trials must be conducted to support the health claims associated with plant-based fermented beverages. Additional research is needed to optimize the production and commercial feasibility of bioactive compounds from plant-based fermented beverages.

## Figures and Tables

**Table 2:** Health benefits of plant-based fermented beverages associated with bioactive components.

Plant-Based Fermented Beverage	Identification of Bioactive Compounds	In Vivo Model	Dose and Duration	Health Benefits	Reference
Soy (*Glycine max*)	Aglycone isoflavones	Rats fed a high-fat diet	10 mL per kg body weight/daily/6 weeks	Antihyperlipidemic	[125]
Soy (*Glycine max*)	N.D.	Type 2 diabetic kidney patients	200 mL per patient/daily/8 weeks	Antidiabetic	[126]
Soy (*Glycine max*)	Aglycone isoflavones and vitamins	Rats fed a hypercaloric high-fat, high-fructose diet	10 mL per kg body weight/daily/90 days	Antidiabetic and anti-obesity	[127]
Soy (*Glycine max*)	Aglycone isoflavones	Murine model of aging induced by D-galactose	0.2 mL per animal/daily/8 weeks	Antioxidant and anti-aging	[128]
Oat (*Avena sativa*)	N.D.	Celiac patients	200 mL per patient/daily/6 months	Antihyperlipidemic	[129]
Oat (*Avena sativa*)	Polyphenols and GABA	Rat model with diabetes induced by streptozotocin	7 mL per animal/daily/6 weeks	Antidiabetic and hypolipidemic	[130]
Rice (*Oryza sativa*)	GABA, α-tocopherol, and polyphenols	Rat colitis model	150 mg dissolved in PBS per kg body weight/daily/10 days	Antioxidant	[131]
Rice (*Oryza sativa*)	Polyphenols	Stroke-prone, spontaneously hypertensive rats	40 mg beverage per kg body weight/16 h fasting	Antihypertensive	[132]
Sweet cucumber (*Solanum muricatum*)	GABA	Spontaneously hypertensive rats	2.5 mL per animal/daily/8 weeks	Antihypertensive	[133]
Aksu apple (*Rosaceae*, Malus, *Fuji*)	N.D.	C57BL/6J mice	10 mL beverage per kg body weight/daily/4 weeks	Antidiabetic	[134]

GABA: gamma-aminobutyric acid; N.D.: not determined.

## Data Availability

Not applicable.
